# Validity of self-reported lunch recalls in Swedish school children aged 6–8 years

**DOI:** 10.1186/1475-2891-12-129

**Published:** 2013-09-18

**Authors:** Monica Hunsberger, Pablo Pena, Lauren Lissner, Lisen Grafström, Barbara Vanaelst, Claudia Börnhorst, Valeria Pala, Gabriele Eiben

**Affiliations:** 1 Department of Public Health and Community Medicine, University of Gothenburg, Box 454, Gothenburg 40530, Sweden; 2 Grafströms Mat & Medicin HB, Gottskärsvägen 176 A, Onsala 43994, Sweden; 3 Department of Public Health, Ghent University, De Pintelaan 185, 2 block A, Ghent 9000, Belgium; 4 Department of Statistical Methods in Epidemiology, Leibniz-Institute for Prevention Research and Epidemiology – BIPS GmbH, Bremen, Germany; 5 Epidemiology and Prevention Unit, Fondazione IRCCS Istituto Nazionale dei Tumori, Via Venezian, 1, Milano 20133, Italy

**Keywords:** Dietary intake, 24-hour recall, Child reported dietary intake, Relative validation

## Abstract

**Background:**

Previous studies have suggested that young children are inaccurate reporters of dietary intake. The purpose of this study was to validate a single recall of the previous day’s school lunch reported by 6–8 year old Swedish children and to assess teacher-recorded intake of the same meal in a standardized food journal. An additional research question was whether parents could report their child’s intake of the previous day’s lunch. Subjects constituted a convenience sample from the large, multi-country study Identification and prevention of Dietary- and lifestyle-induced health EFfects In Children and infantS (IDEFICS). Validations of both children’s recalls and teachers’ records were made by comparing results with the duplicate plate reference method.

**Findings:**

Twenty-five children (12 boys/13 girls) aged 6–8 years participated in the validation study at one school in western Sweden. Children were accurate self-reporters of their dietary intake at lunch, with no significant difference between reported and weighed intake (Mean difference (SD): 7(50) kcals, p=0.49). Teachers significantly over-reported intake (Mean difference (SD): 65(79) kcals, p=0.01). For both methods, child-reported and teacher-recorded, correlations with weighed intake were strong (Pearson’s correlations r=0.92, p<0.001 and r=0.83, p<0.001 respectively). Bland-Altman plots showed strong agreement between child-reported and weighed intakes but confirmed systematic differences between teacher-records and weighed intakes. Foods were recalled by children with a food-match rate of 90%. In all cases parents themselves were unable to report on quantities consumed and only four of 25 children had parents with knowledge regarding food items consumed.

**Conclusions:**

Children 6–8 years of age accurately recalled their school lunch intake for one occasion while teachers recorded with less accuracy. Our findings suggest that children as young as six years of age may be better able to report on their dietary intake than previously suggested, at least for one main meal at school. Teacher-recorded intake provides a satisfactory estimate but with greater systematic deviation from the weighed intake. Parents were not able to report on their children’s school lunches consumed on the previous day.

## Background

Dietary assessment methods have differing levels of accuracy, feasibility and costs [1-3] but all are prone to systematic and random reporting errors [4-6]. Reporting errors are particularly an issue in children and adolescents mostly due to age related limitations in cognitive ability [3-6]. Because young children are often unreliable reporters of dietary intake, parent or adult involvement in dietary assessment is generally necessary [7-9]. Reportedly, there is a transition period in cognitive development between 8–12 years of age [3]. This implies that 8 years of age may be a critical turning point in which some children may start to overcome their difficulties in reporting dietary intake.

Depending on the dietary patterns of a given population, children consume a varying proportion of meals at school, and previous studies have explored means of capturing this dietary intake [10-12] in the face of diverse dietary patterns. Swedish school children receive a lunch meal during the school day at no cost. The primary aims of this study are to examine the validity of 6–8 year old Swedish children’s self-reported dietary intake at lunch and teacher-recorded intake of the same meal compared to the reference method. An additional research question was whether a parent could report their child’s intake of the previous day’s lunch.

## Methods

### Study design and participants

The IDEFICS (Identification and prevention of Dietary- and lifestyle-induced health EFfects In Children and infantS) study is a multi-center, prospective cohort study, which includes children aged 2–9 years from 8 European countries. In the period September 2007-June 2008, children from pre- and primary-schools in each of the eight countries participated in the baseline assessment. Parents/guardians provided written informed consent. Data were collected according to standardized operating procedures (S.O.P.) and under adherence to a predefined protocol at each survey center [13]. All survey centers received local ethics approval. In Sweden the Central Ethic Review Board approved this study (#264-07). In most survey centers where children consumed a meal at school, teachers completed school meal forms to cover the period out of parental control. This sub-study is based upon a sample of IDEFICS participants from western Sweden, n=25, (12 boys and 13 girls) aged 6–8 (mean 6.6) years who were interviewed 183–344 days after the IDEFICS baseline survey. These children recalled intake of the previous day’s lunch which teachers had recorded during the meal. In this way we were able to assess the validity of child self-reported intake, as well as validity of teacher-recorded intake, a method used in the main IDEFICS survey.

### SACINA instrument

The SACINA (‘Self Administered Children and Infant Nutrition Assessment’) instrument [14] was developed for assessing children’s dietary intake, based on an instrument previously used with adolescents [15]. SACINA is a computer assisted 24 h dietary recall program with photographs of country-specific foods showing varied portion sizes for estimation and probing questions for usual combinations and often forgotten foods.

### Reference method

In the school lunch study setting, children selected from varied daily offerings of meat/fish/poultry or vegetarian, a salad bar with up to ten vegetables, milk/water and rye crackers; taking as much or as little as they wished. Data were collected using the duplicate plate method by a single unobtrusive research assistant who built a plate(s) that was a duplicate to the child’s own [16]. This observer sat with the children at their meal tables, interacted with the children and teacher, and assembled additional duplicate plates if the child revisited the self-service line. Thus, children were aware of having an additional person at lunch but did not know they were being observed or that they would be interviewed. For this reason it was not considered necessary to schedule reactivity days to accustom the children. Aside from the research assistant being present there was no change to the seating arrangement. Duplicate plates were discreetly set to the side for weighing after the lunch period and to minimize distraction. Any dropped, spilled or exchanged items were included in the estimates of waste. Measurements took place over an extended period beginning in June of 2008 and concluded in December of 2008 with a pause for the summer break. The weight (in grams) for each food item was calculated using an electronic scale (Philips Scale HR2395, capacity 5 kg ±1 g). The portion consumed was calculated by subtracting the plate waste from the amount taken. Calculations of energy intake, protein, fat and carbohydrate are based upon a shared food database built upon the national food databases. The project was overseen by an individual dietitian who specializes in dietary assessment and who followed the S.O.P. for dietary data collection of the IDEFICS study.

### Test method 1

The following day (21–24 hours after) the study dietitian administered an unscheduled SACINA recall of the lunch meal with participating children following the IDEFICS S.O.P. for parental recalls. Children were alone with the dietitian in a quiet room that had a computer on the desk. As described above, SACINA is a computer assisted 24 h dietary recall program with photographs of foods depicting varied portion sizes for estimation. All children made the recall of the previous lunch before consuming lunch on the day of recall.

### Test method 2

In 14 of 25 children, five teachers from five different classrooms recorded food consumption in a standardized food journal during the meal on 14 non-consecutive days after receiving training from the dietitian responsible for the study. Teachers recorded for only one child per meal observation in a standardized food template for: item, quantity consumed, and quantity remaining on the plate which was entered into the SACINA program for analysis by the study dietitian. Eleven records are not included in the study for the following reasons: one contained food items but not amounts and ten were left blank (two of the blank records were from teachers who were assigned two children on the same day). Teachers observed foods consumed while the children were eating and completed estimates of total intake after the children had gone, without clearly specifying multiple visits to the service line or leftover portions but instead estimating overall intake.

### Parents

Parents of each child were telephoned (16 mothers, 3 fathers, 2 unknown, 4 unreached) and asked to recall their child’s intake for the same meal. The same individual study dietitian who performed the recalls with children phoned parents in the evening following their child’s recall at school. During the unscheduled call, parents were asked only if they could recall their child’s dietary intake at lunch on the previous day. The calls followed a standardized script to assure that all parents were asked the same questions in the same manner.

### Anthropometry

Anthropometry was measured at the baseline examination in late 2007 and early 2008 at the child’s school following IDEFICS S.O.P. [13]. Body mass index (BMI) was categorized according to the criteria of the International Obesity Task Force (IOTF) [17].

### Statistical analysis

Normality of distribution was analyzed using the Kolmogorov-Smirnov test. As data were normally distributed, the agreement between the methods (report and recorded v. weighed) was tested using paired *t-*tests. The differences between reported and recorded versus weighed intake were calculated in terms of total energy (kcal), protein (kcal), fat (kcal) and carbohydrates (kcal). Negative values represent underestimation and positive values overestimation of reported and recorded versus weighed intake. Pearson’s correlations assessed the strength of the association between weighed and reported and recorded intake. Agreement between test methods and weighed intake were assessed using the Bland-Altman method [18]. The differences between test methods and weighed intake were plotted against the mean difference respectively adding 95% confidence limits of agreement [19]. All analyses were performed using the statistical software package SPSS for Macintosh (version 20.0, SPSS Inc., Chicago, IL, USA).

The power to detect an energy difference of 50 kcal between the reference method and children’s recalls and teachers’ records are >0.9 and 0.7 respectively and hence satisfactory. The power to detect a difference in macronutrient energy intake ranges from 0.4 to >0.9. The power to detect a correlation of at least 0.60 between reference data at a significance level of α = 0.05 was 0.91 (n=25) for children’s recalls and 0.67 (n=14) for teachers’ records.

### Overall food matches

To examine differences between the measured lunch intake of the 155 weighed food items and the child’s recall, two authors, both dietitians, examined output from both instruments following methods previously published [20,21]. Both the child reported intake and the weighed intake were entered into SACINA for assessment. SACINA calculates total calories and macronutrients based on a comprehensive food composition database [14]. From the SACINA output, foods from the two systems (reference method and child-report) were classified into four mutually exclusive categories: exact matches at the food level, matches at the food-category level (e.g., chopped leaf of lettuce vs. chopped leaf of white cabbage), intrusions (foods reported by the child but not weighed on day of observation) and omissions (foods weighed on the day of observation but not reported in the child recall).

## Results

**Figure 1:**
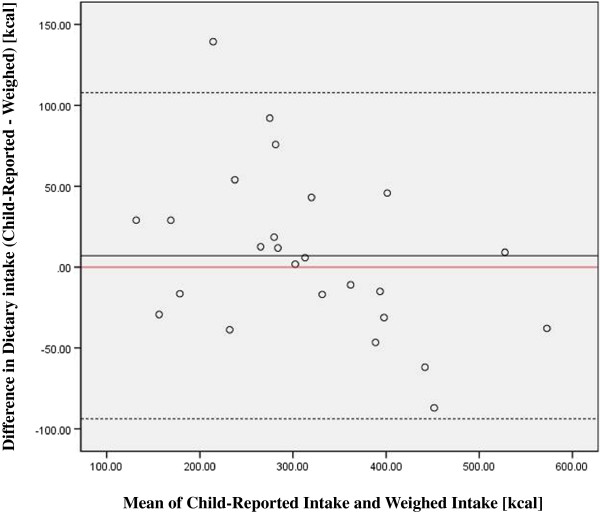
Bland Altman plot depicting differences between the child-report intake and reference method against their mean values, the solid black represents the average difference between the estimate and the weighed food amount (7 kcal child-reported), dotted lines indicate 2 SD from the mean, and the solid red line at zero denotes where the mean would fall if the measurements agreed perfectly.

Weighed caloric intake ranged from 113-579 kcals (average 313 kcals). Differences between child-reported intake, teacher-recorded intake (total energy and macronutrients in kcals) and weighed intake are shown in Table 1 with participant characteristics. The reported intake closely resembled the weighed intake. A paired *t*-test indicated no significant differences between child reported and weighed intake (Mean difference (SD) 7 (50) kcals, p=0.49). Pearson’s correlation showed a strong correlation between children’s recalls and the reference method (r = 0.92, p<.001). Further, agreement by Bland-Altman plot (Figure 1) shows all but one child reported intake within 2 SD of the mean energy. This agreement indicates that these children, 72% under 8 years of age, were good reporters of their intake. Figure 1 also indicates that underreporting by children was larger in cases of large reported energy intakes and child over-reporting was smaller in cases of smaller energy intakes. These findings may be contrasted with those suggesting that children aged seven and younger are not able to accurately report dietary intake [2,3,20,22].

**Table 1:** Participant characteristics, total energy intake by reference method and differences of child-reported and teacher-recorded from reference intake shown in kcals

**Presented by age distribution**				**Total energy and macronutrient reference intake**				**Difference child-reported and weighed intake**				**Difference teacher-record and weighed intake**			
ID	Age	Sex	BMI *	Energy	Protein	Fat	Carb	Energy	Protein	Fat	Carb	Energy	Protein	Fat	Carb
21	6	F	normal	484	56	214	214	−87	−11	−26	−50	35	7	−15	43
22	6	F	normal	464	115	109	239	−62	−19	−20	−23	-	-	-	-
23	6	M	thin	334	83	103	149	−17	−5	−17	5	-	-	-	-
24	6	F	thin	205	38	74	93	54	6	37	11	67	25	−1	43
25	6	M	normal	579	98	236	246	−38	−6	−21	−11	−31	−6	−14	−11
02	7	F	normal	373	57	170	146	46	7	−23	62	−50	−8	−30	−12
04	7	M	normal	366	94	105	167	−11	−7	13	−17	104	33	18	53
05	7	M	normal	397	117	100	180	−15	−23	0	8	-	-	-	-
06	7	M	normal	246	54	45	147	−39	−8	−8	−23	-	-	-	-
07	7	M	normal	149	54	19	77	29	13	5	11	-	-	-	-
08	7	M	normal	252	71	47	134	13	6	3	4	−96	1	−40	−57
11	7	M	thin	170	43	31	96	−29	−8	6	−27	140	17	34	89
12	7	F	normal	226	46	66	115	92	30	−10	72	126	32	31	63
14	7	F	normal	406	79	168	159	−31	−1	−42	12	-	-	-	-
16	7	F	normal	498	74	261	162	9	−19	47	−19	99	−14	123	−10
17	7	M	normal	266	147	18	100	19	−2	1	20	104	67	8	29
19	7	F	normal	307	52	79	176	6	8	25	−27	-	-	-	-
20	7	M	normal	294	41	72	181	43	5	5	33	108	13	9	86
01	8	F	normal	303	46	128	129	5.0	−2	−2	5	-	-	-	-
03	8	M	normal	404	103	113	188	−47	−31	−19	3	-	-	-	-
09	8	M	overweight	113	30	28	56	29	11	13	5	-	-	-	-
10	8	F	normal	269	32	48	188	12	0	11	1	155	25	46	82
13	8	F	normal	238	50	44	143	76	10	2	64	-	-	-	-
15	8	F	thin	141	18	41	82	139	25	63	51	142	26	63	53
18	8	F	normal	182	31	73	78	−16	−3	−7	−6	11	−1	−1	13
Mean difference reported- weighed								7	−1	1	6	65	15	16	32
Median difference reported- weighed								6	−2	1	5	101	14	7	42
SD difference reported -weighed								50	14	24	30	79	21	42	43
Paired t-test p-value								0.49	0.71	0.77	0.30	0.00	0.02	0.17	0.01

p ≤ 0.05 = significance level.

*BMI classification according to IOTF (17).

In a sensitivity analyses examining only children younger than eight years (n=18), the results for the whole study were confirmed; paired *t*-tests showed no statistical differences between child report and observed intake. In addition, Pearson correlation was slightly stronger, (r=0.94) when eight year olds were excluded. It may be of practical significance to note this finding since 8 years of age represents a potentially critical cognitive stage [4].

Examining overall food matches, we found that children who consumed 3–11 food items (mean of 6), recalled foods correctly in most cases. Children matched at the food level 90% of the time, ranging from 67%-100% accuracy. Examined by child age group, 6 year olds had 98% accuracy, 7 year olds 91% accuracy, and 8 year olds 86% accuracy in overall food matches. Interestingly, the older children were the least accurate in reporting. Of the 15 total errors made by 11 children (four of the children made two errors), four errors were made at the food category level, e.g., recalling a red pepper when cucumber was observed, six errors were intrusions and five errors were omissions. Our findings demonstrate young children can report on food items with higher accuracy than a previous study which found children age 6–11 years of age correctly reported 72% of food items [23]. The same study also found that parental report was negligibly more accurate than the child’s report; 78% and 72% respectively [23]. When our findings are compared with earlier findings of child self-report of the lunch meal, children aged 6–12 recalled correctly 60.5%-80.6% of the time on average with accuracy improving across four grade levels (grade 1 to grade 4) [24]. Similar to our findings, others have reported that accuracy is better for a single meal than across an entire day [25].

**Figure 2:**
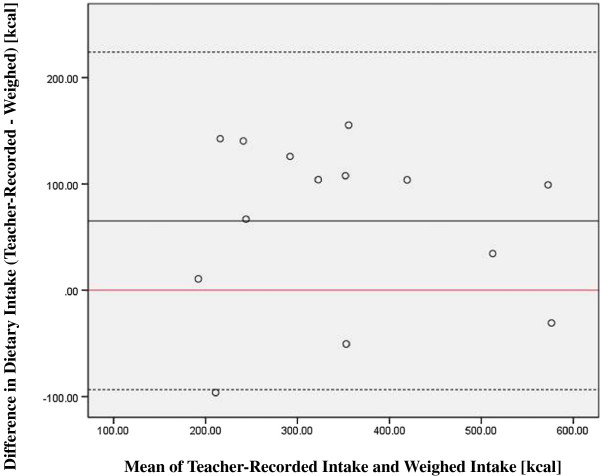
Bland Altman plot depicting differences between the teacher-recorded intake and reference method against their mean values, the solid black represents the average difference between the estimate and the weighed food amount (65 kcal teacher-recorded), dotted lines indicate 2 SD from the mean, and the solid red line at zero denotes where the mean would fall if the measurements agreed perfectly.

Teachers over-reported the children’s dietary intake (n=14) in all but three cases (see Table 1 for energy difference). Approximately half of the over-reporting of calories can be attributed to carbohydrate (33 kcals). A paired *t-*test indicated significant differences between recorded and weighed intake (Mean difference (SD):65(79) kcals, p=0.01). Pearson’s correlation showed a strong association between teachers’ records and the reference method (r = 0.83, p<0.001). Upon inspection by Bland-Altman plot (Figure 2) a systematic disagreement was observed between the methods, although measures are within 2 SD of the mean for all but one subject.

In an additional sensitivity analysis we compared what children served themselves prior to consumption (i.e. disregarding the leftovers) with teachers’ records. A paired *t-*test indicated no significant difference between weighed intake excluding leftovers and teacher recorded intake (Mean difference (SD):42(80) kcals, p=0.08). Teacher-recorded data correlated negligibly better to reference data when leftovers were excluded from the analysis indicated by Pearson’s correlation (r = 0.86, p<0.000). This difference in findings is explained by two subjects who had a large amount of food leftover by reference method, 112 kcals and 141 kcals respectively, which we can only assume was not accounted for by the teacher. All other subjects’ left insignificant amounts of food; from 0 to 30 kcals. This implies that teachers may not have had time to supervise children while making accurate records of food consumed.

Parents (16 mothers, 3 fathers, 2 unrecorded) in 4 of 21 cases were aware of what the child had eaten at school either by menu familiarity or in conversation with their child. In all other instances parents had no knowledge regarding the child’s intake. Not surprisingly, none of the 21 parents reached were able to provide any information on the quantities consumed. Similarly, Livingstone *et al*. and Baranowski *et al.*[4,26] reported low agreement between parental reported dietary intake and reference intake.

A major limitation of this study is its sample size. These results cannot be generalized to larger populations and future research should investigate children’s recall in larger, more diverse populations. Our sample was mainly thin or normal weight, with the exception of one subject, which limits the ability to extrapolate these findings to overweight children who have been shown to under-report energy intake to a larger degree than normal weight subjects [27].

An additional limitation is our validation of only one meal served at school as opposed to a full 24 hour recall. At school the choices may differ compared to a free living situation. These results cannot be generalized to school settings with proportioned servings but may be more relevant for naturalist settings resembling free-living situations. Swedish schools offer a self-service line with notable variety on a buffet table in which students may take as little or as much as they wish. Because school lunches are a main meal provided outside of parental control it is important to identify ways of obtaining this often missing data, our findings may assist others in conducting dietary assessments with young children.

Two additional limitations remain. First, dietary recalls made with both children and later with parents were not audio recorded and therefore we cannot verify that the script in the S.O.P. was followed precisely by the individual study dietitian. Second, parental knowledge may have been better if the time interval had been shorter (i.e. the evening of consumption), although this may have alerted specific children to the interview that would occur the following morning.

Despite these limitations, this study adds value to our knowledge of dietary assessment in young children. It is among the first to examine young children’s’ ability to recall a single meal (lunch) consumed up to 24 hours prior. Eck *et al*. [28] carried out a 24 h recall on the lunch meal consumed by children aged 4–9 years and collected reported intake from the mother alone, the father alone and children reporting with parents. Eck *et al.* found that the group report was more accurate than the individual parental answers, illustrating the value of the child’s contribution. Johnson *et al.*[29] reported that children aged 4–7 reported their energy intake more accurately on a group basis when using multiple 24 h recalls.

It is also possible that the children participating in our study were able to report with a high degree of accuracy due to the fact that the recall was carried out on a single meal as opposed to several meals over a day, as suggested by Baxter and Thompson [25] and Meredith *et al*. [30]. Additionally, the SACINA method offers photos and portion estimation aids which may have contributed to accuracy. In contrast, the lower degree of accuracy found in teachers’ records may be due to external factors, such as being responsible for the care of multiple children at one time. Nevertheless, it is important that children, and to some extent teachers, can report the lunch meal with a high degree of accuracy as this is the most frequently missing meal in parental reported data. Our findings offer future directions for completing missing dietary data using information from children themselves, given that their teachers may not normally have the resources to observe intake and account for leftover portions. Collection of high quality dietary data advances our ability to relate dietary intake to health outcomes.

## Conclusion

Children 6–8 years of age accurately recalled their lunch intake for one occasion while teachers recorded with less accuracy. Our findings suggest that children as young as six years of age may be better able to report on their dietary intake than previously suggested, at least for one main meal at school. Teacher-recorded intake provides a satisfactory estimate but with greater systematic deviation from the weighed intake. Parents were not able to report on their children’s school lunches consumed on the previous day. Future research should investigate potential benefits of child-reported intake in children younger than 8 years of age as it seems that young children may be better reporters than has previously been suggested, at least in case of a single main meal at school.

## Competing interests

None of the authors have any commercial association that would pose a conflict of interest.

## Authors’ contributions

MH analyzed the data and revised the manuscript, PP drafted the manuscript and assisted with data analyses, LL assisted in conceptualizing the study, LG supervised data collection and conducted the 24-hour recalls, BV assisted with data interpretation, CB assisted with data analysis, VP assisted with data interpretation, and GE assisted with conceptualizing the study, supervised data collection, and supervised analysis. All authors read and approved the final manuscript. 
